# A Case Report and Literature Review of Babesiosis-Induced Acute Respiratory Distress Syndrome

**DOI:** 10.1155/2022/4318731

**Published:** 2022-11-12

**Authors:** Philip S. Yune, Iffath Islam, Peter V. Dicpinigaitis, Johanna P. Daily, Louis M. Weiss, Sun O. Park

**Affiliations:** ^1^Division of Infectious Diseases, Department of Medicine, Albert Einstein College of Medicine and Montefiore Medical Center, Bronx, New York, USA; ^2^Department of Medicine, Donald and Barbara Zucker School of Medicine at Hofstra/Northwell, Manhasset, New York, USA; ^3^Division of Critical Care Medicine, Department of Medicine, Albert Einstein College of Medicine and Montefiore Medical Center, Bronx, New York, USA; ^4^Department of Microbiology and Immunology, Albert Einstein College of Medicine and Montefiore Medical Center, Bronx, New York, USA; ^5^Department of Pathology, Albert Einstein College of Medicine and Montefiore Medical Center, Bronx, New York, USA

## Abstract

Babesiosis, a tick-borne protozoan disease, has been increasing in frequency in recent years. Familiarity with presentations of babesiosis is important for clinicians. Acute respiratory distress syndrome (ARDS) is a rarely seen complication of severe babesiosis. In most cases, the patients with babesiosis developed ARDS several days after initiation of antibabesia therapy. We present a unique case of babesiosis without any respiratory symptoms on presentation who developed ARDS within 24 hours of babesiosis treatment initiation. Furthermore, we reviewed published cases of ARDS in babesiosis.

## 1. Introduction

Babesiosis, caused by intraerythrocytic protozoan parasites of the genus *Babesia*, is an infectious disease that is primarily transmitted by ticks, but it can also be transmitted by blood transfusion, organ transplantation, and perinatally [1, 2].

*Babesia* infection can cause a wide range of severity of illnesses ranging from asymptomatic infection to fatal disease [3]. Physical examination findings include fever, pallor, jaundice, retinopathy with splinter hemorrhages and retinal infarcts, splenomegaly, or hepatomegaly [3, 4]. Laboratory abnormalities include hemolytic anemia, thrombocytopenia, and elevated liver enzyme levels [4]. Babesiosis can be complicated by severe anemia, disseminated intravascular coagulation, congestive heart failure, pulmonary edema, renal failure, or splenic rupture [1, 2, 4, 5]. ARDS is a type of noncardiogenic pulmonary edema, characterized by an acute onset of diffuse inflammation responding to an inciting event including infection [6, 7]. Among 15 patients whose sequence of antibabesia therapy and ARDS development was documented, all developed ARDS several days (ranging from 1 to 5 days) after receiving therapy for babesiosis except for one patient from a case report and two patients from a case series who developed ARDS prior to the initiation of therapy (Table 1) [6, 8–13]. We report a case of babesiosis, in which the patient developed respiratory symptoms within 2 hours and ultimately ARDS within 24 hours of antibabesia therapy.

## 2. Case Presentation

A 57-year-old man originally from Bangladesh with no significant medical history other than SARS-CoV-2 pneumonia in February 2021 presented to Montefiore Medical Center, NY in July 2021, complaining of recurrent fever, chills, sweats, poor appetite, generalized weakness, myalgia, headache, left-side abdominal pain, and dark urine for 2 months. Prior to the onset of symptoms, he routinely went fishing in the Hudson Valley, Long Island, and Western New York, US where he would walk through the wooded areas to access fishing sites. Apart from numerous mosquito bites, the patient denied any tick bites or rashes.

**Figure 1:**
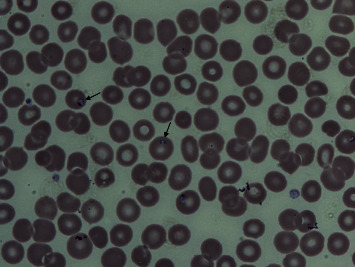
Peripheral blood smear. The arrows indicate intraerythrocytic ring forms (Wright-Giemsa stain). 1000x magnification was used for microscopic examination.

On the initial exam (day 0), the patient appeared to be fatigued but not in any respiratory discomfort with a temperature of 39.5°C, blood pressure (BP) of 117/69 mmHg, heart rate (HR) of 110 beats per minute, respiration rate (RR) of 19 breaths per minute, and oxygen saturation of 96% at room temperature. The rest of the physical exam was normal including a completely normal lung exam. On the initial laboratory evaluation, white blood cell count was 7.9 × 10^3^/*μ*L (normal range, 4.8–10.8) with neutrophils 4 × 10^3^/*μ*L (normal range, 1.8–7.7), lymphocytes 2.3 × 10^3^/*μ*L (normal range, 1–4.8), monocytes 1.4 × 10^3^/*μ*L (normal range, 0.3–0.5), red blood cell count 4.45 × 10^6^/*μ*L (normal range, 4.5–5.9), hemoglobin 10.9 g/dL (normal range, 14.0–17.4), platelet 1.31 × 10^5^/*μ*L (normal range, 1.50–4.00), blood urea nitrogen 9 mg/dL (normal range, 5–20), creatinine 1.0 mg/dL (normal range, <1.30), alanine aminotransferase 37 U/L (normal range, <40), aspartate aminotransferase 44 U/L (normal range, <50), total bilirubin 3.3 mg/dL (normal range, <1.2) with direct bilirubin 1.6 mg/dL (normal range, <0.5), and lactic dehydrogenase 527 U/L (normal range, <240). Human immunodeficiency virus (HIV), hepatitis B, and hepatitis C tests were negative. His abnormal complete blood count triggered a peripheral blood smear exam, which demonstrated intraerythrocytic ring forms consistent with *Babesia* with a parasitemia of 0.7% (Figure 1). The species was later confirmed as *Babesia microti* by a real-time polymerase chain reaction (New York State Department of Health, Wadsworth Center, Albany, NY). A computed tomography scan of the chest, abdomen, and pelvis revealed mild dependent atelectasis and moderate splenomegaly with small infarcts.

On day 1, the infectious diseases consult team recommended treatment for possible coinfection with babesiosis, Lyme disease and anaplasmosis pending full diagnostic testing. The patient received azithromycin 500 mg IV, then doxycycline 100 mg orally (1 hour after azithromycin), and atovaquone 750 mg orally (2.5 hours after azithromycin). The patient then became hypoxic, requiring supplemental oxygen with a flow rate of 3 liters per minute (LPM) via nasal cannula (NC) within 2 hours after the first dose of azithromycin. Within 9 hours of initiation of antibiotics, the patient was noted to have shaking chills and cold extremities with moderate respiratory distress, without lower extremity edema, jugular venous distention, or S3 heart sound. His temperature was 38.5°C, BP 132/108 mmHg, HR 116 beats per minute, RR 26 breaths per minute, and oxygen saturation 86% on supplemental oxygen 3 LPM via NC. Chest X-ray (CXR) showed mild bilateral opacities. The patient required supplemental oxygen therapy with a flow rate of up to 10 LPM via NC, followed by high-flow NC oxygen therapy, then noninvasive ventilation with bilevel-positive airway pressure.

**Figure 2:**
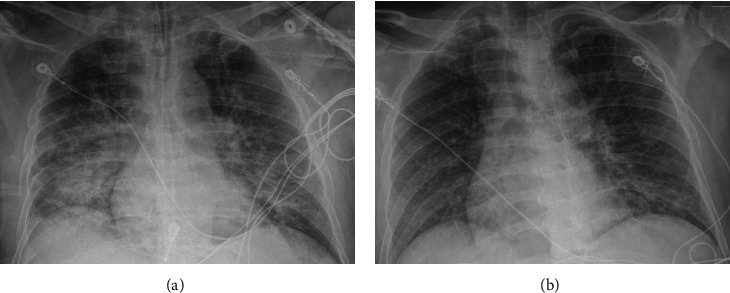
Chest X-rays. Chest X-ray (CXR) which was taken 2.5 hours after intubation (day 2) showed significant diffuse bilateral opacities. (a) CXR next day after extubation (day 7) showed improved bilateral opacities. (b)

On day 2, the patient was intubated within 20.5 hours of antibiotic initiation for his worsening respiratory status and was transferred to the medical intensive care unit. Arterial blood gas 18 hours after intubation was pH 7.41, PaCO_2_ 32 mmHg, PaO_2_ 275 mmHg, on FiO_2_ 100%, positive end-expiratory pressure (PEEP) 10 cmH_2_O. A repeat CXR showed worsening bilateral opacities (Figure 2(a)). The transthoracic echocardiogram was unremarkable with normal left ventricular ejection fraction and wall motion. The Berlin criteria for acute respiratory distress syndrome (ARDS) was met i.e*.,* (a) new respiratory symptoms within 1 week, (b) bilateral opacities, (c) respiratory failure which is not explained by cardiogenic pulmonary edema or cardiac failure, and (d) PaO_2_/FiO_2_ 275 mmHg on PEEP 10 cmH_2_O.

The patient had a recurrent fever for 5 days (days 0 to 4) with his highest daily temperatures ranging from 39.2°C to 39.5°C, then defervesced on day 5. The patient was successfully extubated on day 6. CXR on day 7 showed significant improvement in bilateral opacities (Figure 2(b)). Daily smear exam showed resolution of parasitemia on day 8. Supplemental oxygen and vasopressor support (norepinephrine 2–4 mcg per minute) to maintain mean arterial pressure >65 mmHg (days 4–8) were discontinued on day 9. The patient received a total of 10 days of azithromycin, atovaquone, and doxycycline then was discharged home on day 11.

To rule out coinfection, tests for anaplasmosis and Lyme disease were conducted on day 1, the polymerase chain reaction to detect *Anaplasma phagocytophilum* from the blood sample was negative. Initial two-tiered serologic testing for IgM response to *Borrelia burgdorferi* was positive: Lyme disease Enzyme immunoassay (EIA) index value of 2.99 (index value <0.90 is negative), positive IgM immunoblot with the presence of 23, 39, and 41 kDa protein bands (positive is reactive to ≥2 out of 3 bands), and negative IgG immunoblot with the presence of 41 and 58 kDa protein bands (positive is reactive to ≥5 out of 10 bands). On day 84, repeat two-tiered serologic testing for Lyme disease as an outpatient was negative: Lyme disease EIA index value of 1.08, negative IgM immunoblot with an absence of protein bands, and negative IgG immunoblot with the only presence of 58 kDa protein band.

## 3. Discussion

Over the past 50 years, the incidence of human babesiosis has significantly increased worldwide, especially in the United States (US), where most cases have been reported [1, 4, 14]. Since national surveillance for babesiosis began in 2011, annual reported cases doubled between 2011 (total 1,126) and 2019 (total 2,418) in the US [2] (https://www.cdc.gov/parasites/babesiosis/data-statistics/index.html).

Babesiosis is a zoonotic disease that is transmitted by tick vectors from infected animal reservoirs [1]. Most cases of babesiosis are caused by *B. microti*, which is primarily transmitted by *Ixodes scapularis* ticks from infected white-footed mice from May to October in the Northeastern (NY, MA, CT, NJ, and RI) and upper Midwestern (WI and MN) US [1–4, 14]. Sporadic cases have been caused by *Babesia duncani* in the West and *Babesia divergens*-like organisms in various parts of the US [2]. In Europe, most cases are due to *Babesia divergens* while cases due to *Babesia venatorum* and *B. microti* have been reported [3]. In Asia, cases due to *B. venatorum*, *B. microti*, *Babesia crassa*-like organism, KO1, and XXB/HangZhou have been reported [1].

The mortality rate among hospitalized patients with babesiosis can be approximately 10% with a higher mortality rate among those who are immunocompromised or acquired infection through blood transfusion [1, 3, 15]. While there is no consensus on the definition of severe babesiosis, severe babesiosis has been described as requiring hospitalization >2 weeks, admission to the intensive care unit >2 days, presence of splenic rupture, heart failure, respiratory failure, ARDS, or shock, the need for a red blood cell exchange transfusion, or death [16, 17].

Severe babesiosis typically occurs in patients with one or more of risk factors including asplenia, HIV infection, immunosuppressive therapy, and age >50 years [1, 3, 15]. Our review of ARDS cases reveals similar findings (Table 1). However, patients with advanced age or immunocompromised conditions tend to have comorbidities, which can influence the severity of illness. In a study of 34 hospitalized patients with severe babesiosis in Long Island, NY, the average duration of parasitemia after initiation of treatment was 8.5 days (median, 12 days; range, 3–21 days) and a longer duration of parasitemia for ≥10 days was not associated with complicated babesiosis [5]. High-grade parasitemia is not a requisite for severe babesiosis or ARDS. Greater than 20% of severe babesiosis [5, 6, 17] and 37.5% (6/16 patients) of ARDS caused by babesiosis [6, 8–13] occurred in patients with parasitemia ≤1 percent (Table 1). The studies indicate that the correlation between clinical severity and parasitemia is weak or none [6, 15, 18]. Our patient is another example of severe babesiosis with ARDS that occurred in the setting of low parasitemia. Further studies to shed light on risk factors for severe babesiosis or ARDS are needed.

ARDS is a potentially fatal respiratory condition with a mortality rate of 27–45% [7, 19]. ARDS was the most common complication (20.6%) in the above study from Long Island, NY [5] and was the most common complication (8%) after congestive heart failure (10.9%) in a study of 139 hospitalized patients with babesiosis in the New York State [17]. ARDS evolves from early diffuse alveolar-capillary damage characterized by alveolar-capillary permeability leading to alveolar edema to later fibro-proliferative and fibrotic phases if the patients survive [7, 19]. The Berlin definition of ARDS refined from the 1994 American-European Conference Consensus definition was developed by a panel of experts in 2011, which includes (1) within 1 week of a known clinical insult, or new or worsening respiratory symptoms, (2) bilateral opacities which are not explained by effusions, lobar or lung collapse, or pulmonary nodules, (3) respiratory failure not fully explained by cardiac failure or fluid overload, and (4) moderate to severe impairment of oxygenation as defined by PaO_2_/FiO_2_ (mild, 200 mmHg < PaO_2_/FiO_2_ ≤ 300 mmHg with PEEP or CPAP ≥5 cmH_2_O; moderate, 100 mmHg < PaO_2_/FiO_2_ ≤ 200 mmHg with PEEP ≥5 cmH_2_O; severe, PaO_2_/FiO_2_ ≤ 100 mmHg with PEEP ≥5 cmH_2_O) [7, 19].

The exact mechanism of ARDS caused by babesiosis is unknown. Sequestration of parasitized red blood cells (RBCs) leading to obstruction of the microvasculature of vital organs is the major pathogenesis of *Plasmodium falciparum* and *Babesia bovis* (a causative organism of bovine babesiosis) [1, 20]. However, microvascular sequestration has not been demonstrated in human babesiosis due to *B. microti* [1]. For example, an autopsy of a splenectomized patient with *B. microti* infection with cerebral involvement revealed the absence of erythrocyte sequestration [21]. The release of proinflammatory cytokines in patients with babesiosis may cause cellular damage and vascular leakage leading to ARDS [1]. It is possible that the destruction of babesia organisms after antibabesia therapy triggers a more intense inflammatory response contributing to the development of ARDS [6, 22]. Intriguingly, ARDS caused by malaria often occurs within a few days of starting antimalaria drugs analogous to ARDS caused by babesiosis [20].

Coinfections with additional pathogens that are transmitted by *Ixodes scapularis* including *B. burgdorferi*, *A. phagocytophilum*, *Babesia miyamotoi*, and Powassan virus can occur, resulting in further variation in clinical symptoms [23–25]. Coinfections of *B. burgdorferi* and babesiosis are the most common coinfections in Lyme disease-endemic areas in the US, accounting for more than 80% of coinfections [23–25]. Up to 40% of patients with Lyme disease have concurrent babesiosis, and two-thirds of patients with babesiosis have concurrent Lyme disease [23, 25]. We contemplated a possibility of a coinfection of babesiosis and Lyme disease complicated by Jarisch-Herxheimer reaction to explain our patient's paradoxical worsening of symptoms shortly after the initiation of antibiotic therapy. The Jarisch-Herxheimer reaction is a transient systemic reaction including fever, chills, rigors, headache, hypotension, and worsening rash, which typically occurs within 24 hours after the patients infected with spirochetes receive treatment for such infections [26]. However, ARDS as a result of a Jarisch-Herxheimer reaction associated with Lyme disease treatment has not been described contrary to another spirochetal disease, tick-borne relapsing fever, which could potentially cause ARDS as a result of a Jarisch-Herxheimer reaction [26, 27]. Furthermore, it is unusual for the initial positive IgM bands for Lyme disease to disappear within 3 months if it were a true case of Lyme disease while no additional gain of IgG bands may be explained by early antibiotic therapy [28, 29]. The presence of a transient false positive IgM response to *B. burgdorferi* during active babesiosis has been recently described, which could be due to polyclonal B cell activation triggered by babesiosis [30]. Taken together, we suspect that the initial Lyme disease test was a false positive due to immune activation from the babesiosis in this patient.

In conclusion, ARDS is a rare but major life-threatening complication of severe babesiosis. ARDS occurs more commonly after treatment for babesiosis is initiated. The patient with babesiosis can rapidly develop ARDS despite initial stable respiratory status and low parasitemia burden. Further studies to identify the mechanism and risk factors for babesiosis-induced ARDS are warranted. Clinicians should have a high index of suspicion for concurrent tick-borne diseases especially if the patient is found to have unusual findings for babesiosis alone. Conversely, clinicians should be aware of the possibility of false positive IgM serology test results of other tick-borne diseases associated with active babesiosis.

## Figures and Tables

**Table 1:** Cases and case series of babesiosis-inducedARDS.

	Case	Age (years)/gender	Antibabesia therapy to ARDS (days)	Parasitemia (%)	Died	Comorbidities
Gordon et al.	1	79/F	5	10	Yes	Perforated diverticulitis
Iacopino et al.	2	63/F	2	12	No	Not available
Horowitz et al.	3	70/M	3	2	No	Not available

Boustani et al.	4	65/F	2	15	No	Lyme disease
	5	40/M	1	1	No	HTN, Lyme disease
	6	74/M	Unknown	0.1	No	Lyme disease
Panduranga et al.	7	59/M	N/A	<1	No	HTN, HLD, Afib
Stowell et al.	8	25/F	2	20	No	Splenectomy for HS, seizure, hypothyroidism, depression

Alvarez De Leon et al.	9	36/M	N/A	0.05	No	SSD, HC, cirrhosis
	10	72/F	1	15	Yes	HTN, DM
	11	53/F	4	12.8	No	HTN, UTI
	12	55/M	2	10	No	Splenectomy
	13	54/F	N/A	4	No	Asthma
	14	75/F	4	0.5	No	HTN, CLL
	15	69/M	2	4.7	Yes	HTN, CKD
	16	81/F	1	0.5	Yes	HTN, CAD, asthma

Note. N/A, not applicable as the patient developed ARDS before antibabesia therapy. Abbreviations: ARDS, acute respiratory distress syndrome; F, female; M, male; HTN, hypertension; HLD, hyperlipidemia; Afib, atrial fibrillation; HS, hereditary spherocytosis; SSD, sickle cell disease; HC, hemochromatosis; CLL chronic lymphocytic leukemia; DM, diabetes mellitus; UTI, urinary tract infection; CKD, chronic kidney disease; and CAD, coronary artery disease.

## Data Availability

No data were used to support the findings of this study.
